# LONELINESS AMONG OLDER CAREGIVERS IN THE CALIFORNIA HEALTH INTERVIEW SURVEY

**DOI:** 10.1093/geroni/igae098.1966

**Published:** 2024-12-31

**Authors:** Xiayu Chen, Elizabeth Lydon, Sarah Jones, Vincent F Mathias, Wendy Rogers, Raksha Mudar, Minakshi Raj

**Affiliations:** University of Illinois Urbana-Champaign, Champaign, Illinois, United States; University of Illinois Urbana-Champaign, Champaign, Illinois, United States; University of Illinois Urbana-Champaign, Champaign, Illinois, United States; University of Illinois Urbana-Champaign, Champaign, Illinois, United States; University of Illinois Urbana-Champaign, Champaign, Illinois, United States; University of Illinois Urbana-Champaign, Champaign, Illinois, United States; University of Illinois Urbana-Champaign, Champaign, Illinois, United States

## Abstract

In the U.S., adults aged 65 and above represent the second largest age group among familial caregivers. Navigating dual challenges in caregiving demands and their own aging process may lead to higher risk of social isolation for older caregivers. However, research regarding loneliness among older caregivers remains limited. This study aimed to (a) compare risk factors for loneliness between older caregivers and non-caregivers; (b) evaluate if sociodemographic, health, and caregiving characteristics are related to loneliness among older caregivers; and (c) compare the association between these characteristics and loneliness among older caregivers of persons with dementia versus without dementia. We conducted a secondary data analysis of the 2020 California Health Interview Survey. One-fifth of the older adults (n=7,638) served as caregivers in the past year, and 15% of older caregivers experienced loneliness. Using a series of weighted multivariable logistic regressions, we found that older caregivers were more likely to experience loneliness compared with older non-caregivers (aOR = 1.15, 95% CI: 0.89 - 1.49). Those caring for other older adults had a lower likelihood of loneliness compared with those caring for younger individuals (aOR = 0.48, 95% CI: 0.27 - 0.85). Caregivers of persons with dementia were more likely to experience loneliness compared to those caring for persons without dementia (aOR = 1.67, 95% CI: 1.05 - 2.66). Our analyses revealed that older caregivers, especially those assisting persons with dementia, face a higher risk of loneliness, underscoring the need for targeted support and inclusive caregiving policies.

